# Visualizing Hospital Management Data in R Shiny—A Case Study

**DOI:** 10.3390/healthcare12181846

**Published:** 2024-09-14

**Authors:** Benjamin Voellger, Milica Malesevic-Lepir, Mohamed A. Hafez Abdelrehim, Dalibor Bockelmann

**Affiliations:** 1 Department of Neurosurgery, Klinikum Bad Hersfeld, Seilerweg 29, 36251 Bad Hersfeld, Germany; milica.malesevic@klinikum-hef.de (M.M.-L.); mohamed-hafez.abdelrehim@klinikum-hef.de (M.A.H.A.); 2 Department of Neurosurgery, University Hospital Marburg, Baldinger Str., 35043 Marburg, Germany; 3 Department of Anesthesiology, Intensive Care and Pain Therapy, Klinikum Bad Hersfeld, Seilerweg 29, 36251 Bad Hersfeld, Germany; 4 Stabsstelle Medizinische Strategie und Vernetzung, Universitätsklinikum Freiburg, Breisacher Str. 153, 79110 Freiburg im Breisgau, Germany; dalibor.bockelmann@uniklinik-freiburg.de

**Keywords:** hospital management, neurosurgery, patient satisfaction, performance indicators, spatiotemporal analysis

## Abstract

Objective: There is a demand to make hospital management information beyond basic key performance indicators (KPIs) accessible for clinicians. Methods: We developed an interactive application (IAPP) in R Shiny to visualize such information. We provided the IAPP source code online. As a use case, we recorded basic KPIs (numbers of patients (NPs), reimbursed valuation ratios (RVRs), mean length of stay (LOS)), main diagnoses (MDGNs), main procedures (MPRCs), and catchment area (CA) by district from April 2022 to March 2024 at the index department in central Germany, where a neurotrauma and spinal surgery service was resumed on 1 April 2022. Case mix indexes (CMIs) were calculated. We retrieved information about online-reported patient satisfaction (ORPS) from an online physician rating platform between January 2022 and March 2024. Information on longitudes and latitudes of the index department and neighbouring hospitals was collected. We calculated car travelling isochrones (CTIs) of the hospitals as a proxy variable for accessibility. Chi-square and Fisher’s exact served as statistical tests. Results: During the observation period, the monthly NPs increased from 26 to 43, the RVR showed a 3.96-fold increase, the CMI showed a 2.41-fold increase, and the LOS reached a steady state in the 2nd year after service resumption. CA (*p* = 0.03), MDGNs, and MPRCs diversified. ORPS trended towards better overall evaluation after service resumption (*p* = 0.09). CTI mapping identified a unique market position of the index department. Conclusions: The IAPP makes extended hospital management data accessible to clinicians, can inform other stakeholders in healthcare, and can be tailored to local conditions.

## 1. Introduction

Bad Hersfeld is a city of approximately 31.000 inhabitants in central Germany in the federal state of Hesse [1]. Its history dates back to the 8th century AD, when Lullus founded a Benedictine monastery there [2]. While teaching at the local grammar school, Konrad Duden published a famous dictionary that standardized German spelling in 1880, and it was thenceforth named after him [3]. From 1957, the electronic engineering pioneer Konrad Zuse and his enterprise built some of the world’s first computers in Bad Hersfeld [4].

Effective from 1 April 2022, the author B.V. was appointed as head of the neurosurgical department (“index department”) at Klinikum Bad Hersfeld, Germany (“index hospital”). Supported by one traumatology resident on rotation and two fellow neurosurgeons, he aimed to resume a neurotrauma and spinal surgery service that had initially been established by another team in 2007.

Strengths identified at the index hospital included investment-friendly management, well-established tumor boards, and a well-equipped department of neurology. Hospital controlling provided key performance indicators (KPIs) of strategic [5] value, namely the numbers of patients (NPs), reimbursed valuation ratios (RVRs), the length of stay (LOS), and case mix index (CMI) per department, in a spreadsheet on a monthly basis. We will refer to this set of indicators as “basic KPI”, and we provide a list of abbreviations and acronyms used in this work in Table A1, Appendix A.

As of April 2022, the following weaknesses were found: information on main diagnoses (MDGN), main procedures (MPRC), patient satisfaction, catchment area (CA), and accessibility of the hospital required compilation. The leadership culture had room for improvement beyond addressing a lack of neurosurgical standard operating procedures (SOPs). The index department was not authorized to provide training for residents in neurosurgery.

Similar to the current situation at many other German hospitals [6], there was an ageing workforce with a subsequently increasing shortage of skilled professionals. Technology was, in part, outdated with limited access to it. Operating room (OR) resources and bed capacities were limited.

Opportunities arose from the subsidence of the coronavirus disease 2019 (COVID-19) pandemic in 2022 [7], from demographics with an increasing proportion of elderly patients suffering from degenerative diseases of the spine, from a forthcoming reform of the German healthcare system [8], and a from projected new hospital building adjacent to the existing facilities.

Threats consisted of a highly competitive regional environment with regard to spinal procedures [9]. This is one reason why the department’s reputation had suffered in the years prior to service resumption. The strengths, weaknesses, opportunities, and threats (SWOT) of the index department are summarized in a SWOT analysis chart [10] (Table 1).

In 2024, an interest in compiling and interpreting information beyond basic KPI made us develop an interactive application (IAPP) that depicts access to a given hospital and to selected neighbouring hospitals, as well as CA, basic KPIs, MDGNs, MPRCs, and the online reported patient satisfaction (ORPS) of a given clinical department. Several other applications that provide similar information are either not freely available, do not sufficiently reflect the peculiarities of the German health system, are unable to integrate information obtained from within a given department, or do not allow for reputation monitoring (Table A2 in Appendix B). Here, we explain how to implement an application covering these aspects in R Shiny [11], with data from the index department serving as an example. The geographic information part of the IAPP was inspired by the interactive maps supplementing a recent study on socio-spatial inequities faced by children in German cities [12].

## 2. Materials and Methods

For patients treated at the index department between 1 April 2022 and 31 March 2024, we retrieved NPs, the postal code of main residence, RVRs, LOS, MDGNs, and MPRCs from the hospital information system (HIS) on a monthly basis. Postal codes were converted to information on districts. Data were anonymized before further processing and were collected in comma-separated values (.CSV) files. Table A3, Table A4, Table A5, Table A6 and Table A7 in Appendix C demonstrate outlines of the files. The complete .CSV files are available at the online repository Github [13] (https://github.com/benvoellger/MangoShiny (accessed on 23 August 2024)). CMI was calculated as follows:

CMI = RVR/NP

For the purpose of publication, information on LOS, RVRs and CMIs is provided relative to the respective April 2022 baseline. Districts beyond the contiguous part of the index hospital’s catchment area were excluded from the dataset (Table A3, and [13]) to ensure anonymity. When diagnoses or procedures occurred only once in a particular month, data were omitted to ensure anonymity (Table A5 and Table A6, and [13]).

**Figure 1:**
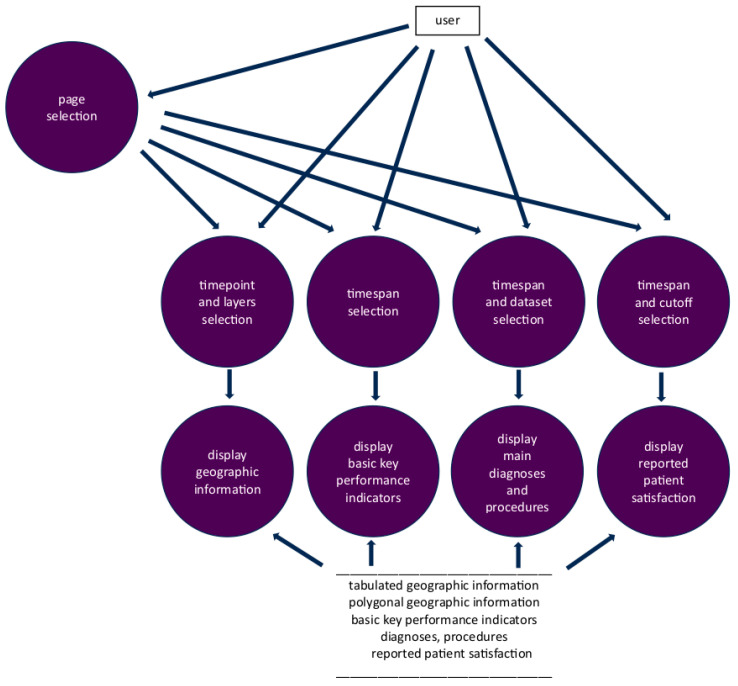
Data flow diagram of the interactive application.

The application was implemented in R Studio version 2023.12.1+402 [14], running R version 4.3.3 [15] on the Mac operating system (OS) Sonoma [16]. Figure 1 depicts the IAPP data flow diagram. The workflow of programming the application is depicted in Supplementary Figure S1. The structure of the application source code is outlined in Supplementary Figure S2. An explanation of the source code is provided as a supplement to this work.

Information on the latitude and longitude of hospital locations was retrieved with the help of Google Maps [17] and was stored in a .CSV file (Table A8 in Appendix C, and [13]). Car travelling isochrones (CTIs, i.e., polygons of locations reachable by car within a specified time limit) were considered a proxy variable for access to the hospitals. CTIs of 30 and 60 min were calculated with the help of Open Route Service [18] (https://openrouteservice.org (accessed on 14 June 2024)), and the resulting polygonal outlines were saved to R data serialization (.RDS) files, which were stored at the online repository [13]. A 3^rd^-party license applies to the use of these shapefiles.

District outlines were downloaded in a .ZIP container with shapefiles (.SHP) from DIVA GIS (https://diva-gis.org (accessed on 10 September 2024)) [19]. The district outline .SHP files are a necessary part of the IAPP and are therefore also deposited at the online repository [13], with another 3^rd^-party license applying to their use.

**Figure 2:**
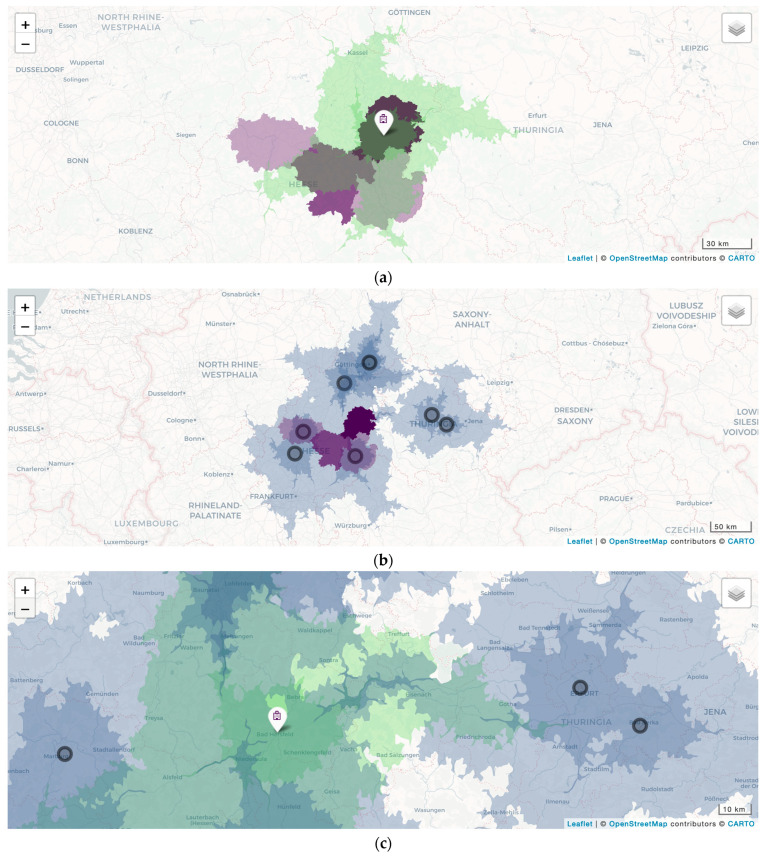
(**a**) Car travelling isochrones (CTIs; 30, 60 min; green; color intensity decreases with travelling time) to the index hospital (white pin) and August 2022 catchment area of the index department by district (purple; color intensity increases with numbers of admissions); (**b**) overlap between index department catchment area by district (purple) and CTIs (30, 60 min; blue; color intensity decreases with travelling time) of competitors (black circles) with equal or higher numbers of beds and levels of service; (**c**) the unique market position (green, without blue overlay) of the index department (white pin) along the Hessian–Thuringian border, with supply gaps to the north and south (map without overlay). The application allows us to zoom, to depict district-wise data by the month, and to toggle layer visibility.

Polygons were rendered with the help of the R leaflet function. They were then layered (Figure 2) onto a smooth tile map, to which another 3^rd^-party license applies.

We recorded overall evaluations from patients treated at the index department, as reported online at the rating platform www.klinikbewertungen.de (accessed on 14 June 2024) [20] (ORPS) between January 2022 and March 2024 in a .CSV file (Table A7, and [13]).

**Figure 3:**
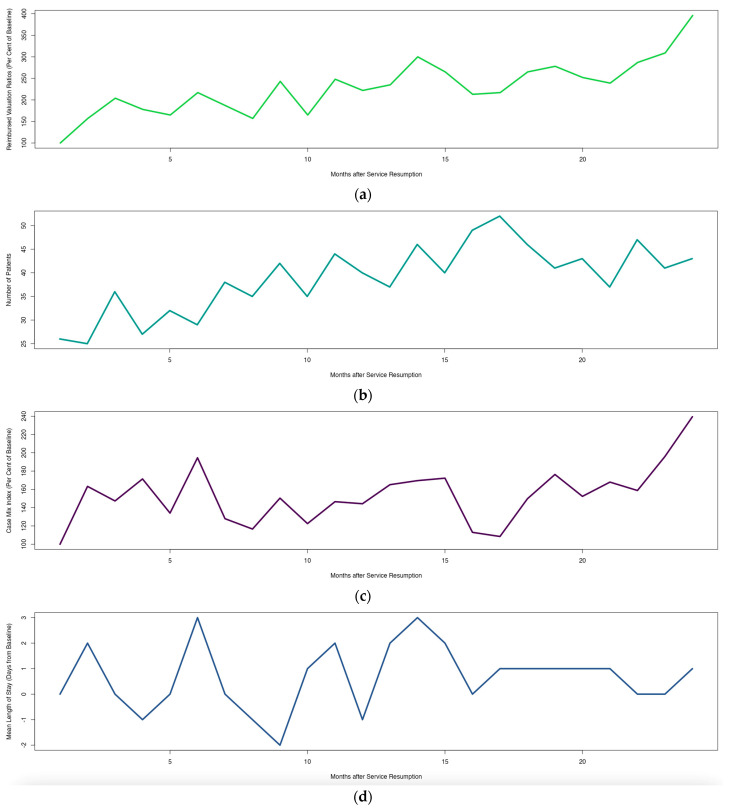
Basic key performance indicators of the index department during the first 2 years after service resumption: (**a**) reimbursed valuation ratios (RVRs); (**b**) number of patients (NPs); (**c**) case mix index (CMI = RVR/NP); (**d**) mean length of stay (LOS). For the purpose of publication, we provide information on RVRs, CMIs and LOS relative to the respective April 2022 baseline (April 2022: 100 per cent of revenue, 0 days from baseline). The application allows us to customize the timespan on the x axis.

**Figure 4:**
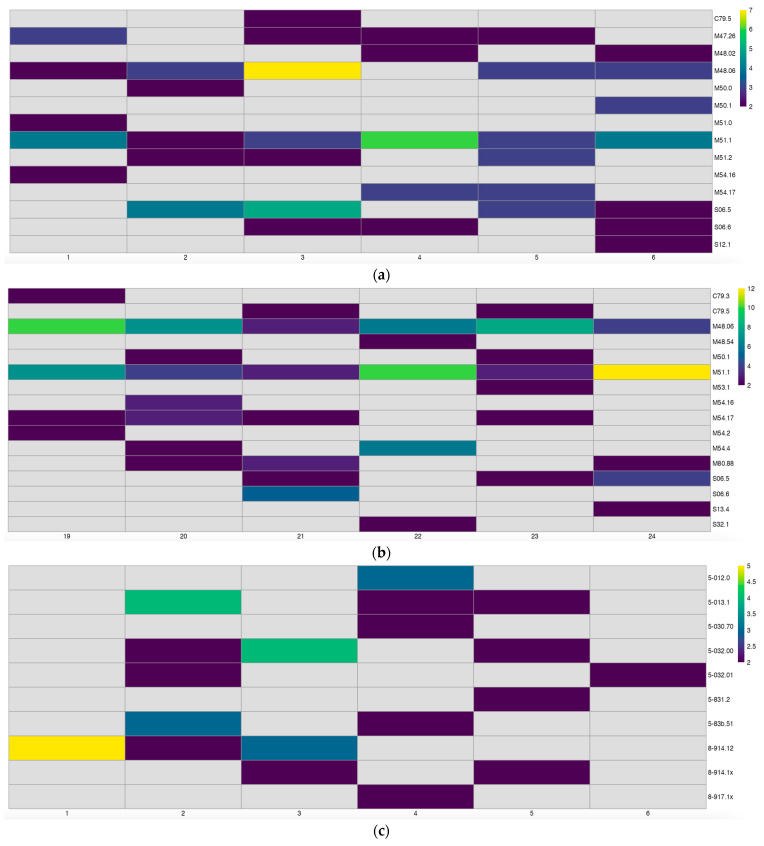

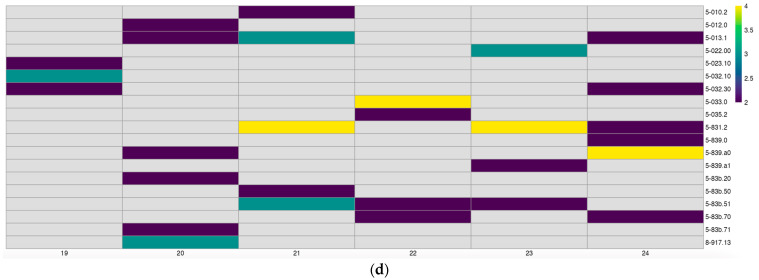
The diversification of further performance indicators of the index department: (**a**) Main diagnoses (MDGNs) according to the 10th version of the German edition of the international classification of diseases and related health problems (ICD-10) at 1–6 months after service resumption; (**b**) MDGNs at 19–24 months after service resumption; (**c**) main procedures (MPRCs) according to the current German version of the international classification of procedures in medicine (ICPM), Operationen- und Prozedurenschlüssel (OPS), at 1–6 months after service resumption; (**d**) MPRCs at 19–24 months after service resumption. Obviously, the numbers of MDGNs and MPRCs increased between the first quarter and the last quarter of the observation period. Colors represent numbers of patients. The application allows us to customize the timespan on the x axis of the heatmaps, with the extension of the y axis changing accordingly and seamlessly.

**Figure 5:**
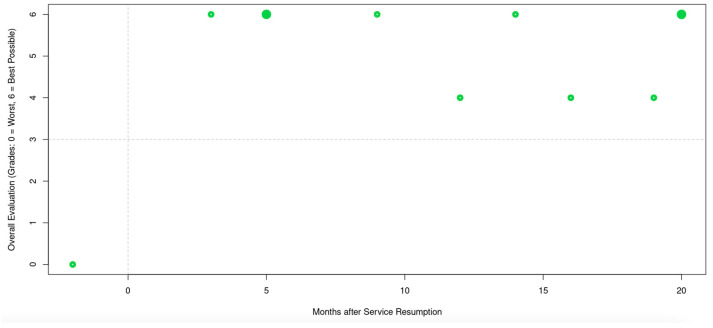
Online reported patient satisfaction. Dashed lines in the figure divide the contingency table cells. Circle diameters increase with evaluation count. Two sliders in the application allow to adjust the positions of the dashed lines along the x and y axes, with Fisher’s exact test *p* value calculated on the fly. A trend towards overall better evaluation after service resumption was observed (Table 2; Fisher’s exact test; *p* = 0.09).

Figure 1 was created with Microsoft Word for Mac v. 16.85.2 [21], then cropped with GIMP 2.10 [22], while Figure 2, Figure 3, Figure 4 and Figure 5 were created with the IAPP [13,23] and then cropped with GIMP 2.10 on the same machine.

Statistical analyses were conducted with the same versions of R and R Studio as above, on the same machine. Fisher’s exact served as statistical test, with a *p* less than 0.05 considered significant.

## 3. Results

### 3.1. The Application

The application was deployed at the Shinyapps server [23] (Figure 2, Figure 3, Figure 4 and Figure 5, https://nc77.shinyapps.io/mangoshiny, accessed on 10 September 2024). Outlines of the .CSV datasheets used in the application are provided in Appendix C of this article. The application source code is provided under the Massachusetts Institute of Technology (MIT) license [24], while 3^rd^-party licenses apply to parts of the deployed geographic information. The complete source code of the application, all necessary .CSV, .RDS and .SHP files, and 3^rd^-party licenses, where applicable, are deposited at Github [13]. The source code and appearance of the IAPP may change over time as they are continuously improved.

At the current stage, the IAPP contains four different pages: a page with a zoomable map and the option to toggle several layers of geoinformation on a monthly base (Figure 2); a page with basic KPI graphs (RVR, NP, CMI = RVR/NP, and LOS) with a customizable timespan (1–24 months, Figure 3); a page with heatmaps of MDGN and MPRC with a customizable timespan (1–24 months, Figure 4); and a page on ORPS with customizable cutoff values for time and overall evaluation, with Fisher’s exact *p* value for the resulting contingency table calculated on the fly (Figure 5).

### 3.2. Use Case: The Index Department

#### 3.2.1. Performance Monitoring

Between April 2022 and March 2024, the monthly NP increased from 26 to 43, monthly RVR showed a 3.96-fold increase, and CMI showed a 2.41-fold increase (Figure 3, and [13,23]). LOS was highly volatile throughout the first year and entered a steady state during the 2nd year after service resumption (Figure 3, and [13,23]).

The catchment area (*p* = 0.03, Figure 2a, Table 3, and [13,23]), MDGN, and MPRC (Figure 4, and [13,23]) diversified during the first two years after service resumption. The catchment area of the index department overlaps with the CTI of neighboring hospitals (Figure 2b, and [13,23]). ORPS trended towards better overall evaluation (*p* = 0.09) after service resumption (Figure 5, Table 2, and [13,23]).

#### 3.2.2. Decision Support

While Figure 3 displays basic KPIs on a 2-year basis, and Figure 4 displays dynamics of MDGN and MPRC on a 6-month basis, a slider in the IAPP allows for customizing the displayed timespans. A clinician may want to use this feature, e.g., to prepare for quarterly discussions with his/her hospital management board, to modify the range of treatments offered, or to develop targeted communication strategies.

The IAPP identified a unique market position of the index hospital along the Hessian–Thuringian federal state border (Figure 2c). Thus, the IAPP facilitates selectively approaching general practitioners whose catchment area includes that particular region.

#### 3.2.3. Beyond Shiny

In spring 2022, neurosurgical tray optimization yielded both a reduction in the number of trays and the number of different instruments. The availability of the magnetic resonance imaging (MRI) scanner outside working hours significantly improved in 2023. A new surgical microscope arrived in July 2023. In 2022 and 2023, authors B.V. and M.A.H.A. developed neurosurgical SOPs. A fixed minimum number of beds at a standard care ward was assigned to the index department in 2023. OR capacity assigned to the index department increased in 2024. These measures probably had an impact on the trends we observed in the IAPP.

The Hessian medical board granted the index department a 2-year training authorization for neurosurgery residents, effective from 1 January 2024. 

## 4. Discussion

In Germany, patients may deliberately choose their doctors. At the beginning of the observation period, the running costs of German hospitals were primarily funded through diagnosis-related group (DRG)-based billing [25]. Different healthcare systems may require the collection and processing of other data in order to measure a given department’s performance adequately.

A reform of the German healthcare system is currently underway, with the aim of rewarding hospitals not only for the realization but also for the provision of healthcare services [8]. The reform will require the coordinated efforts of all stakeholders in healthcare to ensure the optimum allocation of patients and resources [26]. Until the reform bears fruit, and probably also afterwards, information on basic KPIs, patient satisfaction, and the careful monitoring of competitive environments will remain crucial for the successful management of a given clinical department in Germany.

Clinicians, hospital-controlling staff, and healthcare policymakers may modify the IAPP so that it informs routine quarterly discussions at a given hospital, as well as long-term decisions, e.g., in the context of the upcoming reform. For this purpose, the geographic information part of the IAPP may be extended to display any healthcare-related spatial information, such as population density, hospital bed numbers and levels of service, or access to general practitioners.

For the purpose of publication, we provide information on RVR, CMI, and LOS relative to the respective April 2022 baseline. For everyday use inside an organization, we strongly recommend storing all necessary information as absolute numbers in a .CSV file.

Due to the geographic setting of the index hospital—right in the center of Germany with 3 motorways each a 10–15 min car drive away from the emergency room—we considered CTI an appropriate proxy variable for hospital accessibility. With the help of CTI mapping, we identified a unique market position for the index department (Figure 2c), which will be addressed in the near future. Different settings may require other proxy variables for accessibility.

Patients have a variety of choices for how and where to communicate their degree of satisfaction with medical treatment. Our analysis focused on one widely accepted German commercial physician rating website [20]. Since online rating platforms are prone to reporting bias [27,28], reports on dissatisfactory experiences may prevail. On the other hand, a bias may occur when doctors deliberately encourage their most satisfied patients to post rather flattering reports online. In response to negatively biased online evaluation, reputation management, to a certain degree, is probably widespread among clinicians [29]. We do not find this reprehensible, as long as only real treatment reports are published. Although technically feasible in R [15], we do not recommend deploying software for the real-time harvesting of information from online rating platforms.

The advantages of our IAPP are as follows: The software is open-source with the complete source code and the anonymized dataset of the use case deposited at Github [13]. At the current stage, the IAPP is non-commercial while provided under the MIT license, which allows for commercial extension in the future. With a wealth of R libraries at hand, the IAPP is highly customizable. As opposed to numerous competing software bundles (Table A2), the software we developed is available for free. It allows for information from inside and outside a given organization to be available to clinicians within a single application.

The main limitations of our work are as follows: It uses a single-center retrospective approach, focusing on the lean visualization of hospital management data. We designed our IAPP primarily with the still DRG-driven German health system in mind. Underlying data were curated on highly subjective grounds. MDGNs and MPRCs, as obtained through HIS queries, cannot reflect the nuances of the respective treatment. Knowledge of R as a programming language, to a certain degree, is required to customize or extend the IAPP. A commercial extension of the software will require obtaining other, i.e., commercial, 3^rd^-party geoinformation licenses. External sources of information currently deployed with the IAPP, e.g., freely available geoinformation, may change over time. Nonetheless, we think that our IAPP may be helpful in other settings.

## 5. Conclusions

Our IAPP makes extensive hospital management data accessible to clinicians and other stakeholders in healthcare. With knowledge of R as a programming language, the IAPP allows for extensions and it can be tailored to local conditions.

## Figures and Tables

**Table 1:** Analysis of the index department’s strengths, weaknesses, opportunities, and threats (SWOT).

	Positive	Negative
Internal	Strengths	Weaknesses
	monthly updates on basic KPI *, investment-friendly management, tumor boards, department of neurology	need to compile other performance indicators, missing neurosurgical SOPs **, no neurosurgical training authorization, ageing workforce/staff shortage, limited resources (technology, OR ^#^, beds)
External	Opportunities	Threats
	subsidence of the COVID-19 ^##^ pandemic, demographics, forthcoming healthcare reform, funding inquiry for projected new hospital building	competitive environment, suboptimal reputation

* key performance indicators, ** standard operating procedures, ^#^ operating room, ^##^ coronavirus disease 2019.

**Table 2:** Contingency table with data on reported patient satisfaction.

	Overall Evaluation Less Than 4 *	Overall Evaluation 4 * or Higher
Prior to service resumption	1	0
After service resumption	0	10

* 0 = worst, 6 = best possible evaluation; table cells represent numbers of evaluations; online reported patient satisfaction (ORPS) trended towards better overall evaluation after service resumption (Fisher’s exact test; *p* = 0.09).

**Table 3:** Contingency table with data on catchment.

	District of Hersfeld-Rotenburg *	Elsewhere **
Months 1–12 after service resumption	341	61
Months 13–24 after service resumption	410	108

Table entries represent numbers of patients. * hosts the index department; ** refers to the contiguous part of the index department catchment area (CA) beyond the district of Hersfeld-Rotenburg. The proportion of patients admitted from districts other than Hersfeld-Rotenburg was significantly higher during the 2nd year as compared to the 1st year after service resumption (Chi-square; *p* = 0.03).

## Data Availability

The anonymized datasets underlying this work and the complete source code of the application are available online at Github (https://github.com/benvoellger/MangoShiny (accessed on 23 August 2024)).

## Supplementary Materials

The following supporting information can be downloaded at: https://www.mdpi.com/article/10.3390/healthcare12181846/s1, Figure S1. Programming workflow. Figure S2. Structure of the application source code. * comma separated value files; ** shapefiles; *** R data serialization files.

## Appendix A

This appendix contains an overview of abbreviations and acronyms used in this manuscript.

**Table A1:** Explanation of abbreviations and acronyms used in this manuscript.

Abbreviation or Acronym	Full Text
AD	Anno Domini
CA	Catchment area
CMI	Case mix index
COVID-19	Coronavirus disease 2019
CSV	Comma-separated values file
CTI	Car travelling isochrone
DRG	Diagnosis-related group
GIS	Geographic information system
HIS	Hospital information system
IAPP	Interactive application
ICD	International classification of diseases and related health problems
ICPM	International classification of procedures in medicine
KPI	Key performance indicator
LOS	Length of stay
MDGN	Main diagnose
MIT	Massachusetts Institute of Technology
MPRC	Main procedure
MRI	Magnetic resonance imaging
NP	Number of patients
OPS	Operationen- und Prozedurenschlüssel
OR	Operating room
ORPS	Online reported patient satisfaction
OS	Operating system
RDS	R data serialization file
RVR	Reimbursed valuation ratio
SHP	Shapefile
SOP	Standard operating procedure
SWOT	Strengths, weaknesses, opportunities and threats
WHO	World Health Organization
ZIP	Lossless data compression file

## Appendix B

This appendix contains an overview of online available software similar to our IAPP, with a comparison of features.

**Table A2:** The features of our app as compared to those of selected other software.

Service	Ref. nr. *	Processes German Geoinformation	Integrates Internal and External Information	Provides Basic KPI **	Allows for Reputation Monitoring	Available for Free
GeoCare	[30]	No	No	No	No	Yes
Deutsches Krankenhausverzeichnis	[31]	Yes	No	No	No	Yes
WHO *** Accesmod 5	[32]	Yes	No	No	No	Yes
Domo	[33]	Yes	Yes	Yes	Yes	No
Advanced MD	[34]	Unknown	Yes	Yes	Yes	No
Tableau	[35]	Yes	Yes	Yes	Unkown	No
Power BI	[36]	Yes	Yes	Yes	Unkown	No
Our App	[13,23]	Yes	Yes	Yes	Yes	Yes

* reference number; ** key performance indicators, *** World Health Organization.

## Appendix C

This appendix contains outlines of the .CSV data tables used in the application. The complete source code with all .CSV, .RDS and .SHP files can be found at Github [13].

**Table A3:** Catchment area of the index department by district and month. Table cell entries represent numbers of patients.

Month *	183 **	186 ***	…
1	18	1	.
2	21	2	.
3	28	3	.
.	.	.	.
.	.	.	.
.	.	.	.

* month 1 = April 2022; ** geographic information system (GIS) district code 183 refers to Landkreis Hersfeld-Rotenburg, which is where the index hospital is located; *** GIS district code 186 refers to the neighbouring Schwalm-Eder-Kreis.

**Table A4:** Monthly basic key performance indicators of the index department.

Month *	Patients **	valuationRatios **^,##^	meanLOS ^#,##^
1	26	100	0
2	25	157	2
3	36	204	0
.	.	.	.
.	.	.	.
.	.	.	.

* month 1 = April 2022; ** the monthly case mix index (CMI) does not need separate tabulation, as it is calculated, dividing monthly valuation ratios by monthly numbers of patients; ^#^ mean length of stay (LOS) in days; ^##^ for the purpose of publication, valuation ratios and LOS are given relative to the April 2022 baseline (April 2022: 100 per cent of revenue, 0 days from baseline).

**Table A5:** Monthly main diagnoses of patients treated at the index department, according to the 10th version of the German edition of the international classification of diseases and related health problems (ICD-10). Table cell entries represent numbers of patients.

Month *	M48.06 **	M51.1 ***	…
1	2	4	.
2	3	2	.
3	7	3	.
.	.	.	.
.	.	.	.
.	.	.	.

* month 1 = April 2022; ** lumbar spinal stenosis; *** lumbar disc hernia.

**Table A6:** Monthly main procedures of patients treated at the index department, according to the current German version of the international classification of procedures in medicine (ICPM), Operationen- und Prozedurenschlüssel (OPS). Table cell entries represent numbers of patients.

Month *	5-032.00 **	8-914.12 ***	…
1	n/a ^#^	5	.
2	2	2	.
3	4	3	.
.	.	.	.
.	.	.	.
.	.	.	.

* month 1 = April 2022; ** monosegmental dorsal approach to the lumbar spine; *** imaging-assisted lumbar periradicular infiltration; ^#^ not applicable.

**Table A7:** Reported patient satisfaction.

Month *	Evaluation **	Frequency ***
−2	0	1
3	6	1
5	6	2
.	.	.
.	.	.
.	.	.

* month 1 = April 2022; ** 0 = worst, 6 = best possible; *** monthly number of evaluations of the same grade.

**Table A8:** Geographic information.

Loc *	Long **	Lat **	Alt ^#^	Class ^##^
HEF	9.71345	50.87573	Hospital Bad Hersfeld	Index Hospital
GI	8.66635	50.57467	University Hospital Giessen	Neighbouring Hospital (Hesse)
EF	11.01048	50.99336	Hospital Erfurt	Neighbouring Hospital (Other)
.	.	.		.
.	.	.		.
.	.	.		.

* district of hospital location, abbreviated with a car plate code (EF = Erfurt, GI = Giessen, HEF = Hersfeld-Rotenburg); ** longitude (long) and latitude (lat) of the emergency room driveway; ^#^ information presented when hovering over the mapped location with a mouse pointer; ^##^ map layering class of the hospital.
