# Supplementary material for: Visualizing Hospital Management Data in R Shiny—A Case Study
Source: Healthcare (Basel). 2024 Sep 14;12(18):1846. doi: 10.3390/healthcare12181846 (PMC11432085; doi:10.3390/healthcare12181846)
Supplement: Supplementary file 1 [file healthcare-12-01846-s001.zip › suppl_figS2.pdf]

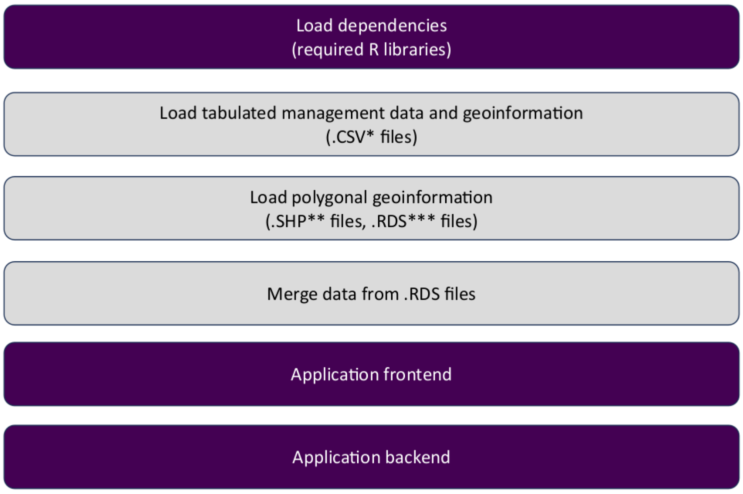

Supplementary figure S2. Structure of the application source code. * comma separated value files; ** shapefiles; *** R data serialization files.
