# Supplementary material for: Visualizing Hospital Management Data in R Shiny—A Case Study
Source: Healthcare (Basel). 2024 Sep 14;12(18):1846. doi: 10.3390/healthcare12181846 (PMC11432085; doi:10.3390/healthcare12181846)
Supplement: Supplementary file 1 [file healthcare-12-01846-s001.zip › supplemental_explanation_of_source_code.pdf]

Supplement to "Visualizing Hospital Management Data in R Shiny – A Case Study" by Voellger et al.

Explanation of the source code of the application

In this supplement, we aim to make the R source code of our application comprehensible. The supplement is tailored to explain how R receives and processes all necessary information to get the application up and running. Commands and operators are explained usually once, while they may recur several times in various contexts in the application source code. See the deposited files at Github (ref. nr. 13 in the publication) for complete source code and an anonymized dataset. This supplement is not meant to be an exhaustive R tutorial.

Comments in R start with a hashtag `(#)`. As with every other programming language, it is recommended to use plenty of meaningful verbose comments in order to make the source code easy to understand and maintain, e.g. by applying subheadings. An example for a meaningful subheading in R is:

```
#load dependencies
```

Oftentimes, R source code depends on routines readily available in precompiled libraries, which must be loaded at the beginning of the program. The syntax to load such a library in R is: `library(libraryNameGoesHere)`. Example:

```
library(shiny)
```

Data processed in R can be stored in .CSV files. In order to load .CSV formatted data into R, commands such as `read.csv()` or `fread()` can be used. The latter comes with the library `data.table`. A source file and the separator used in the source file must be defined. The dataset should be assigned to a variable using the left arrow operator `(<-)`. Data tables can be transformed, i.e., rotated by 90 degrees, using the `t()` function. Examples:

```
catchment_data <- read.csv("./data/catchment_data.csv", sep = ";")

ops_data <- t(fread("./data/ops_data.csv", sep = ";"))
```

External data may be downloaded in R with the command `download.file`. Source link and destination file must be specified. In R, .ZIP containers are unpacked to the current directory using the `unzip` command, which requires a source file to be specified. Example:

```
download.file(
      url = http://biogeo.ucdavis.edu/data/diva/adm/DEU_adm.zip",
      destfile = "./gis_data/germany.zip")

unzip(zipfile = "./gis_data/germany.zip")
```

Shapefiles (.SHP) are widely used in geographic information systems (GIS) applications. Shapefiles contain data on polygonal outlines of geographic entities, such as country borders, stored in a simple features (sf) format. The `sf` library is necessary to deal with simple feature data. The R command to read a shapefile is `read_sf`. A source file must and a destination variable should be specified. Example:

```
german_counties <- read_sf("./gis_data/DEU_adm3.shp")
```

Polygons can also be stored in R data serialization (.RDS) format. We used this file format to store car time travelling range (CTTR) data as obtained from Open Route Service. It is necessary to register with openrouteservice.org first, and to request a token there. The command to produce a polygonal shape is `ors_isochrones()`, which comes with the library `openrouteservice`. It is necessary to specify a vector containing longitude and latitude of the place the CTTR refers to, a travelling profile (e.g.

“`driving-car`”, or “`wheelchair`”), a travelling time range in seconds, the token, and the output format. The syntax to save the polygon in .RDS format is: `saveRDS(yourPolygon, "yourFilename.shape")`. A destination variable should be assigned. Example:

```
travellingTimeHEF1800 <- ors_isochrones(
      c((hospitals$long[hospitals$loc == "HEF"]),
        (hospitals$lat[hospitals$loc == "HEF"])),
      profile = "driving-car",
      range = 1800,
      api_key = "yourTokenGoesHere",
      output = "sf")

saveRDS(travellingTimeHEF1800, " ./gis_data/dtHEF_1800s_car.shape")
```

.RDS files are read with the command `readRDS()` and should be assigned to a variable. Example:

```
travellingTimeHEF1800 <- readRDS("./gis_data/dtHEF_1800s_car.shape")
```

Polygons can be merged using the command `st_union()`. Invalid geometries can be made valid applying the `st_make_valid()` command, and the precision of this operation can be adjusted using `st_set_precision()`. That fix, however, has its limitations. We strongly recommend not to exceed nesting of three `st_union()` calls. Otherwise, invalid polygons may result despite any validation attempt. An example from our application returning a valid result is:

```
travellingTimeOtherHospitalsElsewhere1800 <-
  st_union(
    st_union(
      st_make_valid(st_set_precision(travellingTimeAPD1800, 1e6)),
       st_make_valid(st_set_precision(travellingTimeEF1800, 1e6))),
         st_make_valid(st_set_precision(travellingTimeGOE1800, 1e6)))
```

In R Shiny, the user interface of an application is usually assigned to a variable named `ui`. We decided to implement the user interface using `navbarPage()`:

```
ui <- navbarPage( . . . )
```

Within the user interface, one may specify theme, title, menu items and responsive elements such as sliders for each page as in the following, self-explaining example:

```
theme = bslib::bs_theme(bootswatch = "lux"),
windowTitle = "Hospital Management Data Goes Shiny - A Case Study",
navbarMenu(
  title = "Menu",
  tabPanel("Catchment Area",
    title = "Catchment Area",
    sliderInput("month",
      label = "Month after Service Resumption (1 = April 2022):”,
      value = 5, min = 1, max = 24,
      width = 500),
  leafletOutput("map"),
  textOutput("map_legend")
),
```

In a tab panel, figures can be rendered using `plot.Output()`. Radio button triggered conditional figures can be called using `plot.Output()` nested inside a `conditionalPanel()`, with the latter responding to the state of the radio buttons as in the following example:

```
radioButtons("dgn_or_proc",
  label = "",
  choices = c("Diagnoses", "Procedures"), selected = "Diagnoses",
  inline = TRUE),
conditionalPanel(
  condition = "input.dgn_or_proc == 'Diagnoses'",
  plotOutput("icdmap")
  ),
conditionalPanel(
  condition = "input.dgn_or_proc == 'Procedures'",
  plotOutput("opsmap")
  )
```

In R Shiny, the backend of the application is usually assigned to a variable named `server`:

```
server <- function(input, output, session){ . . . }
```

Within the backend, a variable can be made responsive to input from the user interface by declaring the variable `reactive()` to this very input, for example:

```
mo <- reactive(input$month)
```

will make the value of the backend variable `mo` change according to the input “`month`” from the `sliderInput` on the catchment area page of the user interface (see above).

The R pipeline operator `(%>%)` is a very powerful tool. While the obvious use of the operator is to hand over the return value from one function as an input value to the next function, `(%>%)` comes with a hidden, quite decent extra functionality: A `(%>%)` pipelined function will simply be bypassed when a switch inside the function is turned off. Thus, the `(%>%)` operator can be exploited to align complex items which can be dynamically added to or removed from a page in R Shiny. Thereby, a `(%>%)` pipeline enables layering in R Shiny GIS applications:

```
m <- mapview()@map %>%
  leaflet(german_counties) %>%
  setView(lng = (hospitals$long[hospitals$loc == "HEF"]),
      lat = (hospitals$lat[hospitals$loc == "HEF"]), zoom = 7) %>%
  addProviderTiles("Stadia.AlidadeSmooth") %>%
  addScaleBar(position = "bottomright",
      options = scaleBarOptions(imperial = FALSE))
```

In the example above, the variable `m` contains the basic map layout. `leaflet()` is the map rendering function, `setView()` defines a starting point and a zoom level, `addProviderTiles()` adds basic map content with a theme, and `addScaleBar()`, with the parameter `imperial` turned off, adds a metric scale bar. A dynamic pipeline can be developed from here as follows:

```
output$map <- renderLeaflet({
for (i in (2:(ncol(catchment_data)-1))) {
m <- addPolygons(
  group = "Index Hospital Catchment Area",
  m,
  data = (german_counties$geometry[german_counties$ID_3 ==
catchment_data[1, i]]),
  color = ("#440154FF"), opacity = 0,
```

```
    fillOpacity = catchment_data[mo()+1, i]/3, smooth = 0.1)
  }
  m %>%
  addPolygons(
    group = "Neighbouring Hospitals (Hesse)",
    data = travellingTimeOtherHospitalsHesse1800,
    color ="#39568CFF", opacity = 0.3, weight = 0.3, fillOpacity = 0.3
  ) %>%
```

Of note, assigning a certain polygon to a `group` of polygons allows to switch polygon layers on and off further downstream:

```
  addLayersControl(
    overlayGroups = c(
      "Index Hospital",
      "Index Hospital Catchment Area",
      "Neighbouring Hospitals (Hesse)",
      "Neighbouring Hospitals (Other)"),
      options = layersControlOptions(
        collapsed = TRUE,
        position = "topright")
  ) %>%
```

`addAwesomeMarkers()` and `addCircleMarkers()` are there to mark longitudes and latitudes of the hospitals.

`renderText({“yorTextHere”})` renders a text.

The `plot()` function plots graphs. Variables for x and y axes are handed over at the beginning. `xlab = ”. . . ”` and `ylab = “. . . “` define axis labels, `xlim = c(lower_end, upper_end)` limits the x axis, `col = ”#yourHexCodeHere”` defines the color of the graph, `lwd` is for line thickness, `type` is for the type of the line (continuous, dashed, and so on).

The `renderPlot()` function is used for the heatmaps with information on diagnoses and procedures. The `as.matrix()` function extracts information from the `icd_data` table according to the defined timespan `t1():t2()`. `colnames()` turns the selected timespan into x axis labeling. `mat[rowSums(is.na(mat)) != ncol(mat),]` is there to ignore lines when they have no entries throughout the selected timespan. `color = viridis(((t2()-t1())*(nrow(mat)))+24)` adjusts the palette.

`fisher.test(yourContingencyTableHere)$p.value` returns the p value of Fisher’s exact test for a given contingency table. `round(varName, 2)` rounds a given variable to two decimal places. `paste(“yourTextHere”, varName)` combines a sequence of a string and a variable to a new string.
