# Supplementary figures and images for: Visualizing Hospital Management Data in R Shiny—A Case Study
Source: Healthcare (Basel). 2024 Sep 14;12(18):1846. doi: 10.3390/healthcare12181846 (PMC11432085; doi:10.3390/healthcare12181846)

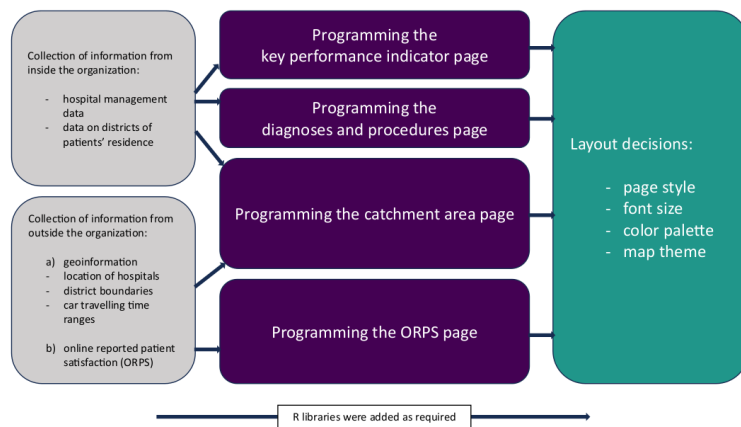

**Supplementary figure S1.** Programming workflow.

Supplement: Supplementary file 1 [file healthcare-12-01846-s001.zip › suppl_figS1.pdf]
